# Presence of active AKT in the nucleus upon adipocyte differentiation of 3T3-L1 cells

**DOI:** 10.17912/micropub.biology.001140

**Published:** 2024-02-29

**Authors:** Marianne Goris, Rhîan G. Jacobsen, Aurélia E. Lewis

**Affiliations:** 1 Department of Biological Sciences, University of Bergen, Bergen, Vestland, Norway

## Abstract

AKT is an essential player in the phosphoinositide 3-kinase (PI3K) signalling pathway. Although the mechanisms of its action are well understood at the plasma membrane, AKT can also be found in the nucleus. In adipocytes, this pathway is activated during the process of adipogenesis and solicits both plasma membrane and nuclear AKT activity. However, the endogenous presence of active AKT in the nucleus during adipogenesis has not been shown. Here, we show that the levels of active AKT phosphorylated at Ser-473 increase rapidly after the induction of differentiation in 3T3-L1 cells, both in the cytoplasm and in the nucleus, and tend to remain elevated over the course of differentiation. In conclusion, these results support the notion that nuclear AKT plays an important role in this process.

**Figure 1. Temporal and spatial activation of AKT upon differentiation of 3T3-L1 cells:**
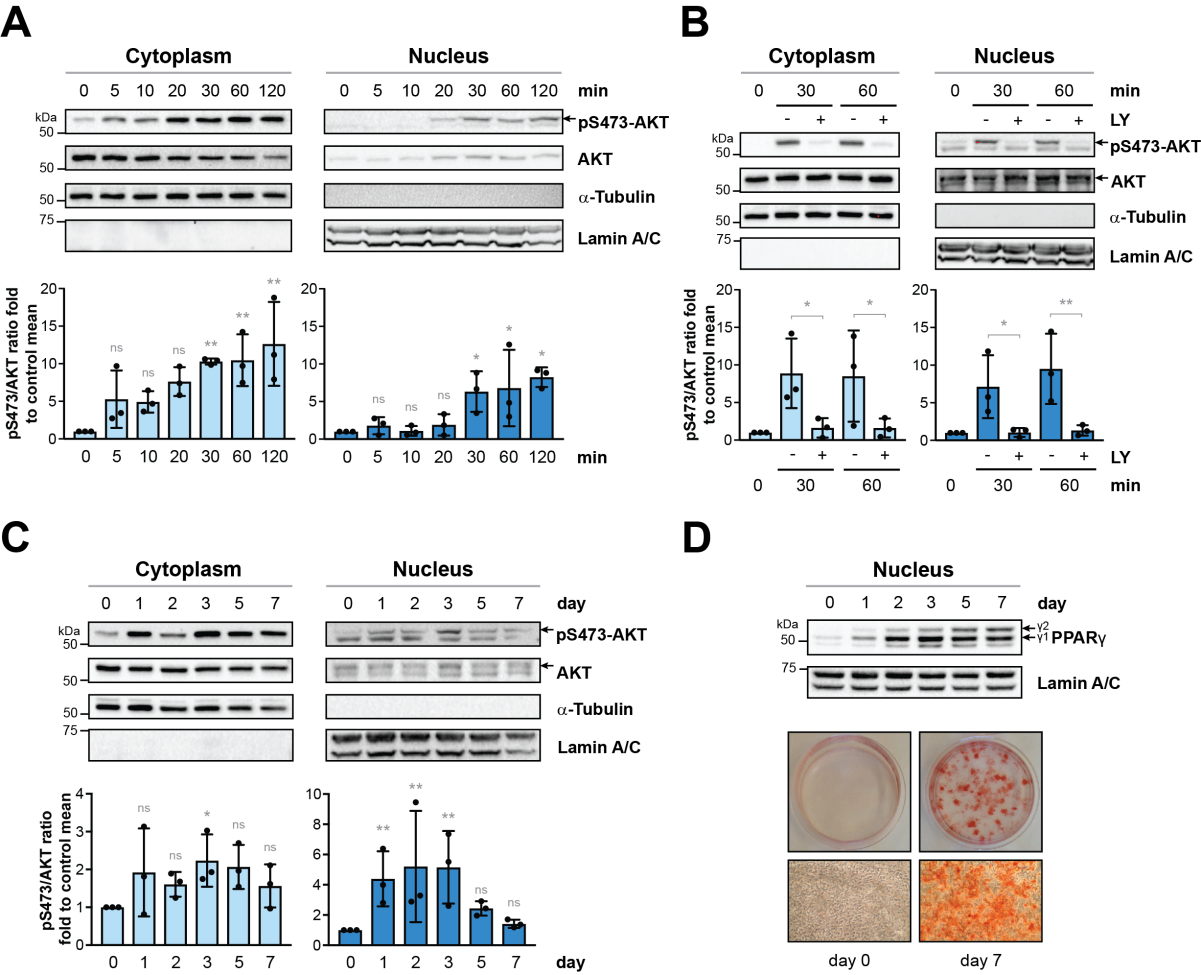
3T3-L1 cells were grown until confluence and two days post-confluent 3T3-L1 cells (time: 0) were induced using a differentiation cocktail containing 500 μM IBMX, 250 nM dexamethasone and 5 μg/ml insulin ( **A** ) for 5 to 120 min, ( **B** ) for 30 or 60 min in the absence (-) or presence (+) of 20 μM of PI3K inhibitor LY294002 (LY) or ( **C-D** ) for 1 to 7 days. **Upper panels A-D:** Cytoplasmic and nuclear fractions from 3T3-L1 cells were then resolved by SDS-PAGE and analysed by Western immunoblotting using indicated antibodies. α-tubulin (cytoplasmic) and lamin A/C (nuclear) were used as fractionation purity markers. **Lower panels** **A-C** : Densitometric analyses of the pSer-473-AKT/AKT ratios compared to two days post-confluent undifferentiated cells (time: 0) from 3 biological experiments + SDs. * *P* ≤ 0.05, ** *P* ≤ 0.01, ns (not significant) (non-parametric, followed by an uncorrected Dunn’s test for panels A and C, One-way analysis of variance (ANOVA) followed by a Fisher’s LSD test for panel B). **Lower panel** **D** : Oil red O staining of triglycerides from fixed 3T3-L1 cells at two days post-confluence (day 0) or at day 7 of differentiation (day 7). Representative photos of 35 mm plates (top) and microscopy images 10x (bottom) are shown.

## Description

The serine/threonine protein kinase AKT (also known as protein kinase B (PKB)) is a central node in the phosphoinositide 3-kinase (PI3K) signalling pathway, which plays important roles in cell proliferation, survival and metabolism (Manning & Toker, 2017) . The mechanism of action of the pathway is well understood following receptor activation at the plasma membrane, where the activation of class I PI3K generates phosphatidylinositol 3,4,5-triphosphate (PtdIns(3,4,5) *P*_3_ ), which in turn recruits the serine/threonine protein kinases AKT and 3-phosphoinositide-dependent protein kinase 1 (PDK1) (Bilanges et al *.* , 2019). AKT is subsequently activated by phosphorylation on Thr-308 and Ser-473 (AKT1) by PDK1 and mTOR complex 2 (mTORC2) respectively (Manning & Toker, 2017) . Active AKT can phosphorylate a myriad of downstream targets triggering a host of cellular effects in different cell types (Manning & Toker, 2017) . In addition to its well-known functions in the cytoplasm, AKT has substrates located in the nucleus and its active form has been detected in the nucleus upon cell stimulation in different cell types (Martelli et al., 2012; Gupta et al., 2022; Morovicz et al., 2022) . For example, an increase in the presence of phosphorylated AKT (pAKT) or AKT activity has been documented within minutes in the nucleus in response to a variety of factors, such as insulin (Ye et al., 2011) , insulin-like growth factor-I (IGF-I) (Borgatti et al., 2000; Leinninger et al., 2004; Wang & Brattain, 2006) , platelet-derived growth factor (PDGF) (Borgatti et al., 2000) , nerve growth factor (NGF) (Borgatti et al., 2003; Xuan Nguyen et al., 2006) and erythropoietin (Missiroli et al., 2009) . Nevertheless, the mechanism leading to the presence of AKT in its active form in the nucleus is still unclear (discussed in Martelli et al. (2012) and Sugiyama et al. (2019)), but in response to stimuli, some studies suggest that prior phosphorylation precedes AKT translocation to the nucleus (Xuan Nguyen et al., 2006; Missiroli et al., 2009) , and others indicate that phosphorylation of AKT can occur within the nucleus itself (Wang & Brattain, 2006) .

The PI3K-AKT pathway plays a crucial role in adipogenesis, as pharmacological inhibition of the pathway blocks adipocyte differentiation (Tomiyama et al., 1995; Xia & Serrero, 1999; Xu & Liao, 2004; Yu et al., 2008; Kim et al., 2009; Jacobsen et al., 2016) and the expression of constitutively active AKT was shown to cause spontaneous differentiation into adipocytes (Kohn et al., 1996; Magun et al., 1996) . Furthermore, an elegant study by Maiuri et al described the generation of molecular tools to inhibit AKT activity in distinct sub-cellular compartments (Maiuri et al., 2010) . Using these tools, they demonstrated that both plasma membrane and nuclear localised AKT are required for terminal differentiation but by contributing to different necessary steps (Maiuri et al., 2010) . The presence of active AKT in the nucleus during this process has however not been shown. In this report, we therefore sought to systematically characterise the temporal presence of active AKT in the nucleus during adipocyte differentiation using the mouse preadipocyte cell line 3T3-L1 cells (Green & Kehinde, 1974) . These cells can be induced to differentiate into fat-laden adipocytes using a cocktail consisting of insulin, 3-isobutyl-1-methylxanthine (IBMX) and dexamethasone (Ruiz-Ojeda et al., 2016) . This cocktail triggers the activation of transcription factors, including CCAAT/enhancer-binding protein (C/EBP) family members and peroxisome proliferation-activated receptor-γ (PPARγ), which culminates in the expression of genes promoting lipogenesis (Tang & Lane, 2012) .

We assessed the subcellular localisation of AKT after the induction of differentiation during both short-term ( Figure 1A ) and long-term ( Figure 1C ) stimulation by detecting the levels of total AKT and pSer-473-AKT in cytoplasmic and nuclear fractions by Western immunoblotting. Fractionation purity was verified using α-tubulin and lamin A/C as cytoplasmic and nuclear markers respectively ( Figure 1A -D). Short-term stimulation with the differentiation cocktail led to a rapid increase in the levels of pSer-473-AKT in the cytoplasm compared to uninduced two days post-confluent cells (labelled as 0 min), with levels tending to increase from as early as 5 min and continuing up to 120 min post-induction ( Figure 1A ). In the nucleus, the levels of pSer-473-AKT significantly increased from 30 min post-induction and remained elevated at 120 min ( Figure 1A ). A double band was detected for pSer-473-AKT in the nucleus, however only the upper band was responsive to inhibition with the pan-PI3K inhibitor LY294002 ( Figure 1B ). Levels of pSer-473-AKT were more varied during long-term stimulation of differentiation in both compartments ( Figure 1C ) but increased from day 1 and overall tended to remain above the level of uninduced two days post-confluent cells (day 0). Total AKT was detected in both untreated and treated post confluent cells over both the short- and long-term stimulations ( Figure 1A, C ). In addition, treatment with the pan-PI3K inhibitor did not prevent the nuclear presence of AKT following stimulation ( Figure 1B ). These observations may hence indicate the possibility of the local activation of AKT within the nuclear compartment upon differentiation. Progression of differentiation over 7 days of stimulation was demonstrated by detecting increasing levels of PPARγ in the nucleus from day 1, consistently with our previous study (Jacobsen et al., 2016) ( Figure 1D ), and by the accumulation of triglycerides which was visualised using oil red O staining on day 7. In summary, these results show an increase in the level of active AKT both in the cytoplasm and nucleus of 3T3-L1 cells upon short- and long-term induction of differentiation. This study hence complements well the findings by Maiuri et al. (2010), who showed that the specific nuclear inhibition of AKT plays a role in the process of differentiation of 3T3-L1 cells.

## Methods

**3T3-L1 cell culture and differentiation**

3T3-L1 cells were kindly provided by Lise Madsen (University of Copenhagen, Denmark). 3T3-L1 cells were cultured in Dulbecco´s modified Eagles medium (DMEM) supplemented with 10% calf serum and 100 units/ml penicillin and 100 µg/ml streptomycin (cultivation medium) at 37 ^o^ C in a 5% CO _2_ incubator and maintained at below 90% confluence. To induce differentiation, 3T3-L1 cells were first grown until they reached confluence and were kept in cultivation medium for two more days (labelled 0 min or day 0 in short- or long-term induction respectively). Cells were then incubated with DMEM supplemented with 10% FBS and 100 units/ml penicillin and 100 µg/ml streptomycin (FBS medium) containing a differentiation cocktail of 500 μM IBMX (I7018, Sigma-Aldrich), 250 nM dexamethasone (D4902, Sigma-Aldrich) and 5 μg/ml insulin (I6634, Sigma-Aldrich). Cells were harvested for cell fractionation at different time points after the addition of the differentiation cocktail. In the experiments requiring the addition of PI3K inhibitor LY294002 (L9908, Sigma-Aldrich), two days post-confluent 3T3-L1 cells were pre-incubated with 20 μM LY294002 (or corresponding volume of DMSO) for 40 min before the addition of the differentiation cocktail containing either LY294002 or DMSO. For long-term differentiation, two days post-confluent 3T3-L1 cells were first incubated in the differentiation cocktail until day 2. The differentiation cocktail was then replaced with FBS medium containing 5 μg/ml insulin and incubated until day 4. After day 4, cells were maintained in FBS medium. Cells were harvested for cell fractionation up to day 7 of differentiation.

**Cell fractionation into nuclear and cytoplasmic fractions**

The cell fractionation protocol is adapted from a method by O'Carroll et al. (2009). All steps were performed on ice with ice-cold buffers. Cells seeded on 100 mm plates were first washed twice in PBS (pH 7.2) and rinsed briefly with hypotonic buffer (10 mM Tris-HCl pH 7.8, 1 % Igepal). 3T3-L1 cells were then scrapped into 500 μl of hypotonic buffer containing 1 mM DTT, 5 mM NaF, 2 mM Na _3_ VO _4_ and 1x mammalian protease inhibitor cocktail (mPIC) (Sigma-Aldrich) and incubated 3 min on ice. 500 μl MilliQ water was added, and the cells were incubated for a further 3 min on ice before being passed 8 times through a 23-gauge needle. Cell lysates were then centrifuged at 400 g for 4 min at 4 ^o^ C. The cytoplasmic fraction (supernatant) was further centrifuged at 600 g for 5 min at 4 ^o^ C to remove nuclear contamination. Isolated nuclei (pellet) were washed in 1 ml wash buffer (10 mM Tris-HCl pH 7.5, 2 mM MgCl _2_ ) and centrifuged at 600 g for 4 min at 4 ^o^ C, the wash step was then repeated. Nuclei were resuspended in RIPA buffer (50 mM Tris-HCl pH 8.0, 0.5 % sodium deoxycholate, 150 mM NaCl, 1 % Igepal, 0.1 % SDS) supplemented with 5 mM NaF, 2 mM Na _3_ VO _4_ and 1x mPIC. Nuclear fractions were further sonicated in a cold ultrasonic bath for 1 min and incubated on ice for 10 min before being centrifuged at 13000 rpm for 5 min at 4 ^o^ C, the supernatant was retained. Protein concentration for both fractions was then determined using Pierce BCA reagent (Thermo Fisher Scientific).

**SDS-PAGE and Western immunoblotting**

Equal amount of proteins (40 μg cytoplasm, 70 or 115 μg nucleus) obtained from cytoplasmic or nuclear fractions were resolved by SDS-PAGE, transferred to nitrocellulose membranes and then blocked in 7% skimmed milk. Antibody and blocking solutions were prepared in PBS-T (PBS pH 7.4, 0.05 % Tween-20). Blots were incubated with primary antibodies overnight at 4 ^o^ C or for 1 h at room temperature before being washed and further incubated with secondary antibodies conjugated to HRP for 1 h at room temperature. Membranes were washed and bands were visualised by enhanced chemiluminescence with SuperSignal West Pico or Femto Chemiluminescent Substrate (Thermo Fisher Scientific) using a BioRad ChemiDoc Xrs+. To strip membranes for further immunoblotting, membranes were first incubated for 20 min at room temperature in Restore Western Blot Stripping Buffer (Thermo Fisher Scientific) before re-blocking. If required, membranes were probed with the corresponding secondary antibody to detect any remaining signal.

Band densities were assessed using ImageJ (NIH, Bethesda, MD). Statistical analyses were performed using the software GraphPad Prism version 7.0e for Mac, using one-way ANOVA followed by the indicated test in the figure legend.

**Oil Red O staining**

Two days post-confluent 3T3-L1 cells (day 0) or 3T3-L1 cells on day 7 of differentiation (day 7) were washed twice with PBS and fixed in 3.7% paraformaldehyde (PFA) in PBS pH 7.4 solution for 15 min at room temperature. A solution of 0.3% Oil Red O (O0625, Sigma-Aldrich) in 60% isopropanol was used to stain cells for 1 hour at room temperature. Cell plates were washed 5 times using MilliQ water and left to dry at room temperature.

## Reagents

**Table:** 

Antibody target	**Supplier**	**Catalogue number**	**Source species**	**Dilution used**
AKT total (pan)	Cell Signaling Technology	#2920	Mouse – monoclonal	1:2000 (cyto) / 1:1000 (nuc)
Phospho-AKT (Ser473) (pSer-473-AKT)	Cell Signaling Technology	#9271	Rabbit - polyclonal	1:1000 (cyto) / 1:500 (nuc)
Lamin A/C	Santa Cruz Biotechnology, Inc	sc-376248	Mouse - monoclonal	1:10,000
PPARγ	Invitrogen	#419300	Mouse - monoclonal	1:5000
α-tubulin	Sigma-Aldrich	T5168	Mouse - monoclonal	1:10,000
Anti-mouse IgG (H+L) - HRP	Invitrogen	G-21040	Goat - polyclonal	1:10,000
Anti-rabbit IgG (H+L) - HRP	Invitrogen	G-21234	Goat - polyclonal	1:10,000
